# Genetic diversity, disease resistance, and environmental adaptation of *Arachis duranensis *L.: New insights from landscape genomics

**DOI:** 10.1371/journal.pone.0299992

**Published:** 2024-04-16

**Authors:** Alicia N. Massa, Victor S. Sobolev, Paola C. Faustinelli, Shyamalrau P. Tallury, H. Thomas Stalker, Marshall C. Lamb, Renee S. Arias

**Affiliations:** 1 National Peanut Research Laboratory, USDA-ARS, Dawson, Georgia, United States of America; 2 Plant Genetic Resources Conservation Unit, USDA-ARS, Griffin, Georgia, United States of America; 3 Department of Crop and Soil Sciences, North Carolina State University, Raleigh, North Carolina, United States of America; Institute for Biological Research, University of Belgrade, SERBIA

## Abstract

The genetic diversity that exists in natural populations of *Arachis duranensis*, the wild diploid donor of the A subgenome of cultivated tetraploid peanut, has the potential to improve crop adaptability, resilience to major pests and diseases, and drought tolerance. Despite its potential value for peanut improvement, limited research has been focused on the association between allelic variation, environmental factors, and response to early (ELS) and late leaf spot (LLS) diseases. The present study implemented a landscape genomics approach to gain a better understanding of the genetic variability of *A*. *duranensis* represented in the *ex-situ* peanut germplasm collection maintained at the U.S. Department of Agriculture, which spans the entire geographic range of the species in its center of origin in South America. A set of 2810 single nucleotide polymorphism (SNP) markers allowed a high-resolution genome-wide characterization of natural populations. The analysis of population structure showed a complex pattern of genetic diversity with five putative groups. The incorporation of bioclimatic variables for genotype-environment associations, using the latent factor mixed model (LFMM2) method, provided insights into the genomic signatures of environmental adaptation, and led to the identification of SNP loci whose allele frequencies were correlated with elevation, temperature, and precipitation-related variables (*q* < 0.05). The LFMM2 analysis for ELS and LLS detected candidate SNPs and genomic regions on chromosomes A02, A03, A04, A06, and A08. These findings highlight the importance of the application of landscape genomics in *ex situ* collections of peanut and other crop wild relatives to effectively identify favorable alleles and germplasm for incorporation into breeding programs. We report new sources of *A*. *duranensis* germplasm harboring adaptive allelic variation, which have the potential to be utilized in introgression breeding for a single or multiple environmental factors, as well as for resistance to leaf spot diseases.

## Introduction

Cultivated peanut (*Arachis hypogaea* L.) is a self-pollinated allotetraploid (AABB genome; 2*n* = 4*x* = 40) originated by natural hybridization of two wild diploid species, *A*. *duranensis* Krapov. & W. C. Greg. (AA genome; 2*n* = 2*x* = 20) and *A*. *ipäensis* Krapov. & W. C. Greg. (BB genome; 2*n* = 2*x* = 20), followed by a single, or a few spontaneous polyploidization events less than 10,000 years ago [1]. These rare and relatively recent evolutionary events created a genetic diversity bottleneck and isolated the tetraploid peanut from its diploid wild relatives [2–4]. Consequently, all modern cultivated forms are characterized by a narrow genetic base and low levels of natural genetic diversity for critical abiotic and biotic stresses.

The intraspecific genetic diversity and variability for disease and insect resistance, make *A*. *duranensis* a valuable resource for peanut improvement and genetic studies. Early molecular characterization based on isozymes, restriction fragment length polymorphism (RFLP), and simple sequence repeat (SSR) markers showed a high level of polymorphic loci among accessions collected from a vast region of the species geographic range [4–8]. High levels of intraspecific variation were also reported for seed storage proteins [9].

*Arachis duranensis* has been reported as a source of resistance to major diseases and insect pests including peanut rust (*Puccinia arachidis*) [10], late leaf spot (*Nothopassalora personata* (syn. *Cercosporidium personatum* (Berk. & Curt.) Deighton) [10], peanut stunt virus (PSV) [11], potato leafhopper (PLH) [12], armyworm (*Spodoptera* spp.) [13], and aflatoxin accumulation by *Aspergillus* species [14–16]. In addition to insect and disease resistance, recent studies on gene expression and functional genetics have showed several characteristics in *A*. *duranensis* accessions that are associated with tolerance to abiotic stresses, including drought, salinity, and extreme temperatures [17–20].

Early leaf spot (ELS) caused by *Passalora arachidicola* (syn. *Cercospora arachidicola* Hori) and late (LLS) leaf spot caused by *Nothopassolora personata* (Berk and M.A. Curtis) U. Braun, C. Nakash., Videira, and Crous (syn. *Cercosporidium personatum*), are major foliar diseases of peanut worldwide. Currently, there is only a moderate level of ELS and LLS resistance in cultivated peanut, consequently, an aggressive fungicide control program is necessary to prevent severe defoliation and yield losses, which may exceed 50% [21]. However, high levels of resistance or immunity to leaf spots have been identified in wild diploid species, including *A*. *cardenasii* Krapov. & W.C. Gregory, *A*. *diogoi* Hoehne, and *A*. *stenosperma* Krapov. & W.C. Gregory [22–27]. A limited number of accessions of *A*. *duranensis* have been evaluated for resistance to ELS and LLS, most of which have been conducted only for LLS and under controlled environmental conditions or detached leaf assays [28–31]. Therefore, relationships between genetic diversity and disease level, impact of environmental factors, and responses to ELS and LLS diseases in the wild progenitor of cultivated peanut merit further investigation.

This study reports the genetic characterization of 33 accessions of *A*. *duranensis* from the USDA peanut germplasm collection representing the entire geographic range of the species in its center of origin in South America (https://npgsweb.ars-grin.gov). The use of SNP markers allowed for a genome-wide characterization of natural populations. The availability of *A*. *duranensis* reference genomes (https://www.peanutbase.org) [2] enabled the incorporation of the positional information of SNPs in association analyses. Landscape genomic analyses provided insights into signatures of environmental adaptation and led to the identification of SNP loci whose allele frequencies are correlated with leaf spot disease incidence and the climatic and spatial distribution of the species. We report new sources of *A*. *duranensis* germplasm that have the potential to be utilized in introgression breeding for abiotic stress tolerance and resistance to leaf spot diseases.

## Materials and methods

### Plant material

**Fig 1:**
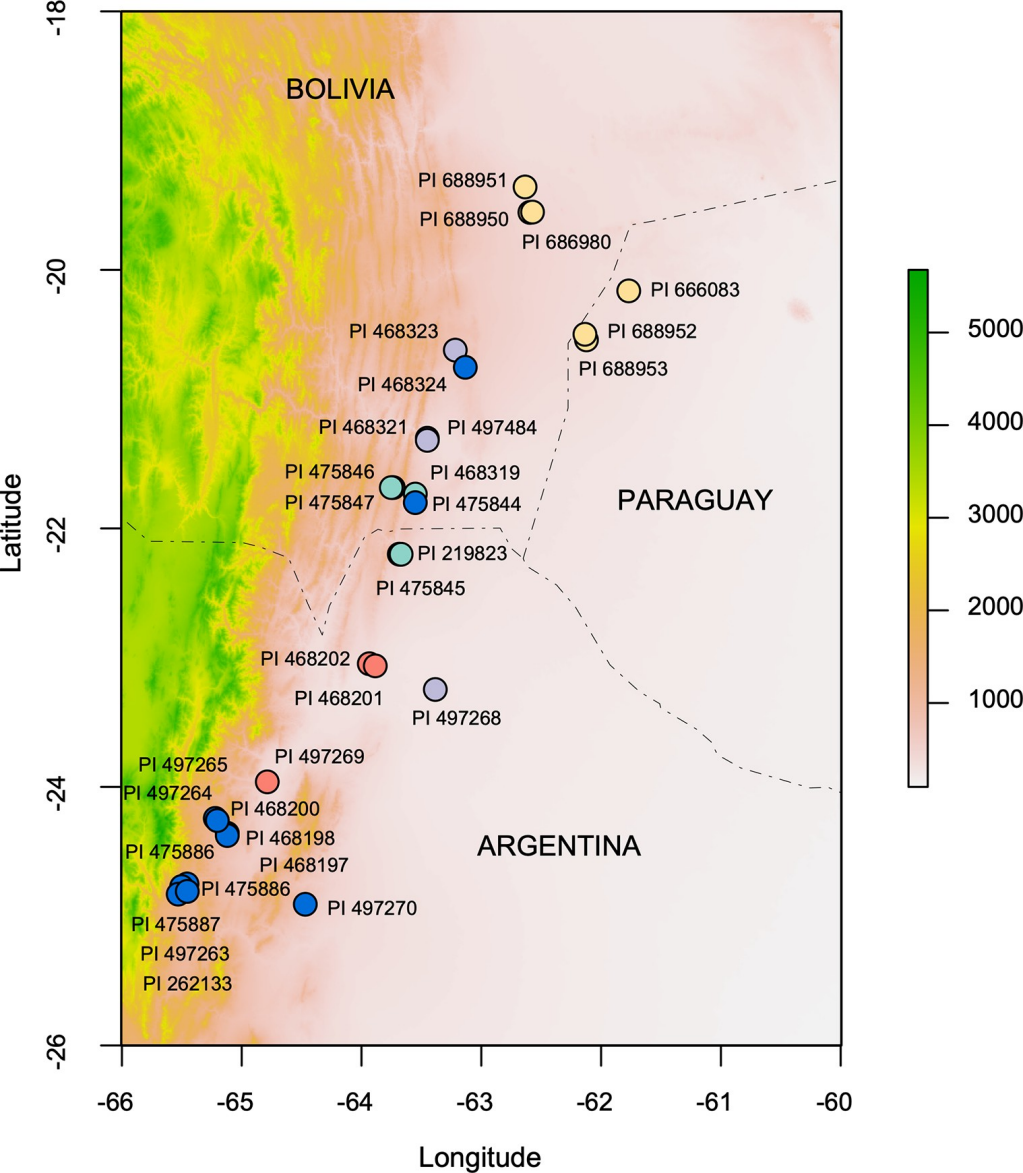
Elevation map with the geographic location of *A*. *duranensis* accessions. Colored circles correspond to the five populations identified by the sparse non-negative matrix factorization sNMF method (Fig 2). Color scale bar indicates meters above sea level. The map was generated with the *raster* R package [32] using the ETOPO2 data with a 15 arc-second resolution [33].

We analyzed 33 accessions of *A*. *duranensis* from the U.S. Department of Agriculture, Agricultural Research Service, National Plant Germplasm System (NPGS), Plant Genetic Resources Conservation Unit peanut (*Arachis*) collection in Griffin, GA (Table 1). For this study, the large leaf (l.l.) and short leaf (s.l.) type from accession PI 262133 (GKP 10038) were treated as different entries [7]. The 33 accessions covered more than 75% of the original collection sites in South America. They extend from -19.35 to -24.82 latitude and from -61.77 to -65.53 longitude, spanning Paraguay, Bolivia, and Argentina (Fig 1). This area covers two of the Level III Ecoregions of Central and South America (http://ecologicalregions.info/htm/sa_eco.htm): a) the Northern Dry Chaco (22.1.1.) and b) the Southern Yungas (18.3.2). The Gran Chaco (22.1) is described as a vast alluvial/colluvial nearly flat plain, with the north area (Northern Dry Chaco, 22.1.1) characterized by a drier and warmer climate than the south area (Southern Dry Chaco, 22.1.2). While the Yungas ecoregion is characterized by warm, wet, forested, species-rich, and windward mountain slopes, with drier valleys in the Southern Yungas (18.3.2). Numbers in parenthesis refer to the ecoregions codes as described (http://ecologicalregions.info/htm/sa_eco.htm). Elevation of the collection sites ranged from 250 to 1300 meters above sea level (Table 1).

**Table 1:** Accessions of *A*. *duranensis* used in this study listed in group order.

Accession ID	Sample name	Latitude	Longitude	Elevation (m)	Origin	Group (Fig 2)	Genotype (Fig 2)
PI 468201	GKBSPSc 30065	-23.066667	-63.883333	350	Argentina, Salta	1	1–1,1–2
PI 468202	GKBSPSc 30067	-23.050000	-63.933333	330	Argentina, Salta	1	1–3,1–4
PI 497269	KSSc 38905	-23.966700	-64.783300	450	Argentina, Jujuy	1	1–5
PI 497484	KSSc 38902	-21.316667	-63.450000	450	Bolivia, Tarija	2	2–1
PI 468321	GKBSPSc 30075	-21.300000	-63.450000	450	Bolivia, Tarija	2	2–2,2–3
PI 497268	KSSc 38904	-23.250000	-63.383333	250	Argentina, Salta	2	2–4,2–5
PI 468323	GKBSPSc 30077	-20.616700	-63.216700	500	Bolivia, Chuquisaca	2	2–6,2–7
PI 219823	K 7988	-22.200800	-63.668000	500	Argentina, Salta	3	3–1
PI 475845	GKBSPSc 30070	-21.883333	-63.633333	600	Bolivia, Yacuiba	3	3–2
PI 475846	GKBSPSc 30071	-21.683333	-63.750000	1000	Bolivia, Tarija	3	3–3,3–4
PI 475847	GKBSPSc 30072	-21.683333	-63.733333	870	Bolivia, Tarija	3	3–5,3–6
PI 468319	GKBSPSc 30073	-21.733333	-63.550000	500	Bolivia, Tarija	3	3–7 to 3–10
PI 688950	Mundubi (DEW 1268)	-19.547778	-62.595000	511	Bolivia, Santa Cruz	4	4–1,4–2
PI 686980	DEW 1270	-19.545556	-62.573333	520	Bolivia, Santa Cruz	4	4–3,4–4
PI 688951	DEW 1274	-19.350556	-62.635278	435	Bolivia, Santa Cruz	4	4–5,4–6
PI 688952	WiSVgJsQ 1506-W	-20.537222	-62.123889	350	Paraguay, Boquerón	4	4–7,4–8
PI 688953	WiSVgJsQ 1507	-20.493889	-62.136389	370	Paraguay, Boquerón	4	4–9,4–10
PI 666083	WiSVg 1510-A	-20.156944	-61.771389	285	Paraguay, Boquerón	4	4–11,4–12
PI 497264	ScVa 21766	-24.366667	-65.116667	1000	Argentina, Jujuy	5	5–1
PI 468197	GKBSPSc 30060	-24.366667	-65.116667	940	Argentina, Jujuy	5	5–2
PI 262133	GKP 10038 s.l.	-24.838130	-65.525650	1250	Argentina, Salta	5	5–3,5–4
PI 262133	GKP 10038 l.l.	-24.838130	-65.525650	1250	Argentina, Salta	5	5–5,5–6
PI 497265	ScVa 21767	-24.250000	-65.216667	1150	Argentina, Jujuy	5	5–7,5–8
PI 468198	GKBSPSc 30061	-24.266667	-65.200000	1000	Argentina, Jujuy	5	5–9,5–10
PI 475844	GKBSPSc 30069	-21.800000	-63.550000	550	Bolivia, Tarija	5	5–11,5–12
PI 468200	GKBSPSc 30064	-24.383333	-65.116667	940	Argentina, Jujuy	5	5–13,5–14
PI 468324	GKBSPSc 30078	-20.750000	-63.133333	500	Bolivia, Chuquisaca	5	5–15,5–16
PI 475886	KSBScC 36006	-24.758830	-65.452080	1278	Argentina, Salta	5	5–17,5–18
PI 475887	KSSc 36036	-24.779150	-65.497000	1250	Argentina, Salta	5	5–19,5–20
PI 497270	KSSc 38906	-24.916667	-64.466667	430	Argentina, Salta	5	5–21,5–22
PI 497263	ScVa 21764	-24.816700	-65.450000	1000	Argentina, Salta	5	5–23
PI 475884	KSBScC 36004	-24.266700	-65.200000	1100	Argentina, Jujuy	5	5–24
PI 497267	KSSc 38903	-22.200000	-63.683333	500	Argentina, Salta	---	

### Environmental variables

Geographic coordinates of the accession collection sites were used to retrieve 19 bioclimatic variables from the WorldClim 2.1 database at 30 second spatial resolution (~ 1 km^2^) between 1970 and 2000 [34,35] (S1 Table). For accessions without exact coordinates, the geographic position was derived from the associated passport data available at the Germplasm Resources Information Network (GRIN) database (https://npgsweb.ars-grin.gov). The WorldClim 2.1 bioclimatic data were extracted, processed, and plotted using the *raster* R package [32]. The elevation map of the collection sites was generated with *raster* using the ETOPO2 data with a 15 arc-second resolution [33]. To reduce dimensionality among environmental factors, we performed a Principal Component Analysis (PCA) including the 19 bioclimatic variables (BIO1 to BIO19) and elevation, using the *prcomp* function in R version 4.1.1 (R Development Core Team, 2023). The first two principal components (Bio-PC1, Bio-PC2) were chosen for genotype-environment associations.

### Disease evaluation

For ELS and LLS disease evaluation, 10 seeds per accession were planted in the greenhouse at the USDA-ARS National Peanut Research Laboratory (NPRL) in Dawson, GA. After 5–6 weeks, plants were transplanted into the field at Newman (31°47’02"N 84°29’16"W) and Bolton (31°47’35"N 84°30’50"W) farms in Georgia, during 2017 and 2018, respectively. Greenhouse-grown plants were also used to extract DNA for genotyping. The experimental design and screening of the ELS and LLS diseases were conducted as described in Massa et al. [23]. Briefly, screening was conducted under field conditions where plants were exposed to naturally occurring inoculum. Disease severity was evaluated by final defoliation rating (FD), early leaf spot lesion counts (ELC), and late leaf spot lesion counts (LLC). For lesion counts, a full expanded leaf was collected from the fourth node from the end of a lateral branch on four plants in each plot and averaged for each genotype. The number of lesions were examined under stereomicroscope and recorded for ELS and LLS separately. Defoliation rating was performed on whole plots at the end of the growing season using the proportional 1–9 scale, where 1 indicates no defoliation and 9 indicates complete defoliation [36,37]. Final defoliation is an accumulation of both early and late leaf spots at the end of the growing season. Six peanut runner cultivars, three susceptible (‘Georgia-13M’, ‘TUFRunner 511’, ‘Georgia-09B’) and three moderately resistant cultivars (‘Georgia-14N’, ‘Georgia-06G’, ‘TifNVHigh OL’) were used as controls [23]. Statistical differences among genotypes in FD, ELC, and LLC variables were determined by analysis of variance (ANOVA). The Tukey-Kramer test was used to perform pairwise comparisons between genotypes, the latter using the HSD.test function in the *agricolae* R package.

### SNP genotyping and analysis

DNA was extracted from young leaves using the QIAGEN DNeasy Plant Mini Kit (QIAGEN, Germantown, MD, USA), quantified by spectrophotometry (NanoDrop 2000, Thermo Fisher Scientific, Waltham, MA, USA), and adjusted to a concentration of 40 ng/μL. All samples were genotyped with the 48K ‘Axiom_Arachis2’ SNP array (Thermofisher Scientific Inc., Waltham, MA, USA) [38]. Marker quality assessment and SNP calling was conducted with the Axiom Analysis Suite v5.0.1 software, using the Axiom best practices genotyping workflow with option set to diploids (Thermo Fisher Scientific Inc., Waltham, MA, USA). All samples with a Dish (dQC) value ≥ 0.82 and QC call rate ≥ 0.97 were considered to pass the quality control. Genotype data were extracted only from the PolyHighResolution (PHR) category. Additional filtering steps were applied to remove SNPs with > 10% missing data and minor allele frequency (MAF) < 0.02 [39].

### Genetic structure and genotype-environment associations

Genetic structure was assessed using inference of populations as implemented in the R package LEA v3.4.0 [40]. Two statistical methods, sparse non-negative matrix factorization (sNMF) and principal component analysis (PCA) were applied to estimate ancestry coefficients as implemented in the *snmf* and *pca* functions, respectively. The function *snmf* was run with *K* = 1–10 (10 replicates for each *K* value) and *α* = 100. LEA algorithms have proven to be more appropriate than other population structure programs such as ADMIXTURE [41] for ancestry estimates of inbred lineages (selfing species) [42], as is the case of *A*. *duranensis*. Principal component analysis was also conducted using the *adegenet* R package [43].

To identify SNP loci associated with environmental clines and with resistance to leaf spots, we used the latent factor mixed models (LFMMs) [42,44]. This analysis tests for significant associations between SNP allele frequencies and the selected variables after correcting for genetic structure using the *lfmm2* function in the LEA R package with the *lambda* default parameter of 1e-5, and number of factors *K* = 5 [40,45]. Missing genotype data were imputed using the *impute* function in LEA. This imputation algorithm replaces missing genotypes with predicted genotypes based on the population structure inference performed in the *snmf* function of the package [45]. The resulting *p*-values were adjusted to account for multiple testing using the Benjamini-Hochberg method with expected levels of false-discovery rate *q* < 0.05. SNPs with *q <* 0.05 were considered candidate adaptive markers.

## Results and discussion

### SNP genotyping and analysis

Genotyping data using the 48 K Axiom_Arachis2 SNP array were obtained for a set of 58 individuals from 32 accessions of *A*. *duranensis*. For accession PI 497267 only ELS and LLS field data was available. Each accession of *A*. *duranensis* was represented by two plants, except for eight accessions, PI 468197, PI 497264, PI 497484, PI 219823, PI 475845, PI 497269, PI 475884, and PI 497263, for which genotyping data were only available from a single plant (Table 1). For PI 468319, SNP data were obtained from four individuals to account for the genetic variation observed within the accession. After quality filtering to remove SNP loci with more than 10% missing data and MAF < 0.02, a set of 2810 reliable and highly informative SNPs were available for further analyses (S2 Table). SNPs were distributed across the 10 wild peanut chromosomes, providing genome-wide coverage, with an average of 281 SNPs per chromosome, ranging from 212 SNPs on chromosome A07 to 382 SNPs on chromosome A02. The final dataset contained less than 0.1% missing data, with 158 missing genotype calls distributed across 129 SNP loci (S2 Table). Imputation of these small missing data did not affect the output of the LFMM2 association analyses as compared to the same analysis performed after removing the 129 SNP loci (data not shown).

### Population genetic structure

**Fig 2:**
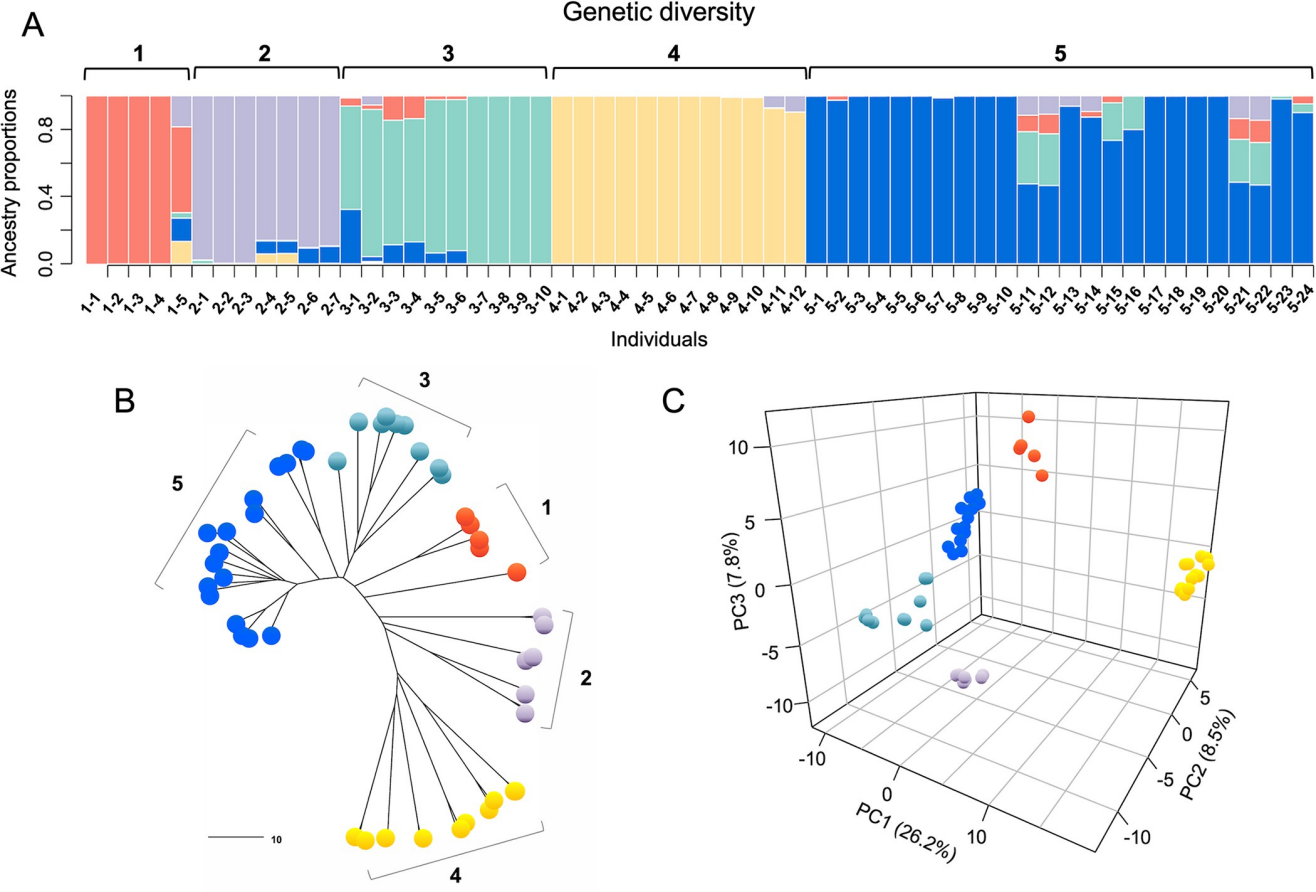
Genetic diversity of *A*. *duranensis* represented in the USDA peanut germplasm collection. (A) Plot of ancestry coefficients obtained from the sparse non-negative matrix factorization analysis (sNMF) for 58 individuals. Each individual genotype is represented by a vertical bar. Numbers 1–5 represent the groups. References for genotype IDs are listed in Table 1. (B) Neighbor-Joining dendrogram based on 2810 SNPs. (C) Population structure based on the first three components of the PCA. Colored circles in (A) and (B) correspond to the five ancestral populations identified by sNMF.

The PCA and admixture sNMF analyses were ran for the complete set of 58 individuals and for a subset of 52 individuals, for which leaf spot disease scores and SNP data were available. The genetic structure inferred from sNMF was rather smooth, with cross-entropy values decreasing slowly as the number of populations increased. However, a subtle “elbow” at *K* = 5, where the values begin to plateau, suggests that five is the number of populations that best explains the genotypic data (Fig 2A). The structure of *K* = 5 was also supported by the PCA (Fig 2C), the phylogenetic relationships (Fig 2B), and the spatial distribution of the accessions (Fig 1). The northern population, represented by six accessions collected in the basin of Parapeti River of Bolivia (Santa Cruz) and West Paraguay sand dunes, within the Northern Dry Chaco ecoregion, contained almost no admixed individuals (cluster 4 in Fig 2). A similar pattern was observed for the southern population with 12 accessions collected in the Southern Yungas of Argentina (Salta and Jujuy) (cluster 5 in Fig 2). Outliers to this group were accessions PI 468324 and PI 475844 from south Bolivia, and accession PI 497270 from Salta, Argentina, with variable proportion from other populations. Three other putative groups, numbered 1 to 3 in Fig 2, were distributed within the geographic range of the species between the northern and southern populations.

Group 1 included two accessions with no admixed individuals (PI 468201, PI 468202) collected near the Rio Seco, in Salta, Argentina, and one accession, PI 497269, collected in Jujuy (Argentina), showing genetic proportions (< 50%) from other three origins. All three accessions in this group are highly similar to the A subgenome of *A*. *hypogaea* [46,47]. With exception of PI 497268, group 2 consisted of three accessions collected in the Pilcomayo River basin of Bolivia, within the Southern Yungas or in the transition to the Northern Dry Chaco. Accession PI 497268 (collected in Salta, Argentina) encompassed admixed individuals with genetic components of northern and southern origin. Population 3 was represented by five accessions distributed in northwest Argentina and south Bolivia, in the basin of the Bermejo River. Some accessions within this group showed admixed individuals with relatively small proportion (< 35%) of genetic components derived from two other origins. The population structure with *K* = 5 was further used as a model to test for association of allele frequencies with environment and disease response gradients.

Results from the analysis of population structure presented here support previous findings documenting the complex patterns of genetic variability of *A*. *duranensis* [2,7,48]. The number of accessions and molecular markers used in the analysis allowed for the detection of admixed genomes and the estimation of their ancestry proportions. With a few exceptions, accessions within each putative group originated from the same geographic area. Those originated from a remote area contained admixed individuals, most likely as the result of natural or human migration long ago, followed by spontaneous hybridization events. This may explain, for example, the unexpected placing of accession PI 475844 which was collected in Tarija, Bolivia, but based on RFLP markers was grouped with the southern accessions [7]. When phylogenetic inference is based on a small number of markers, the position of admixed individuals in a phylogenetic tree is likely to be biased toward one of the ancestral populations. Using 2810 SNP markers, the present study showed that accession PI 475844 encompass admixed individuals with ancestry proportion (approx. 40%) of southern origin (Fig 2).

### Geographic and bioclimatic characterization of the collection sites

**Fig 3:**
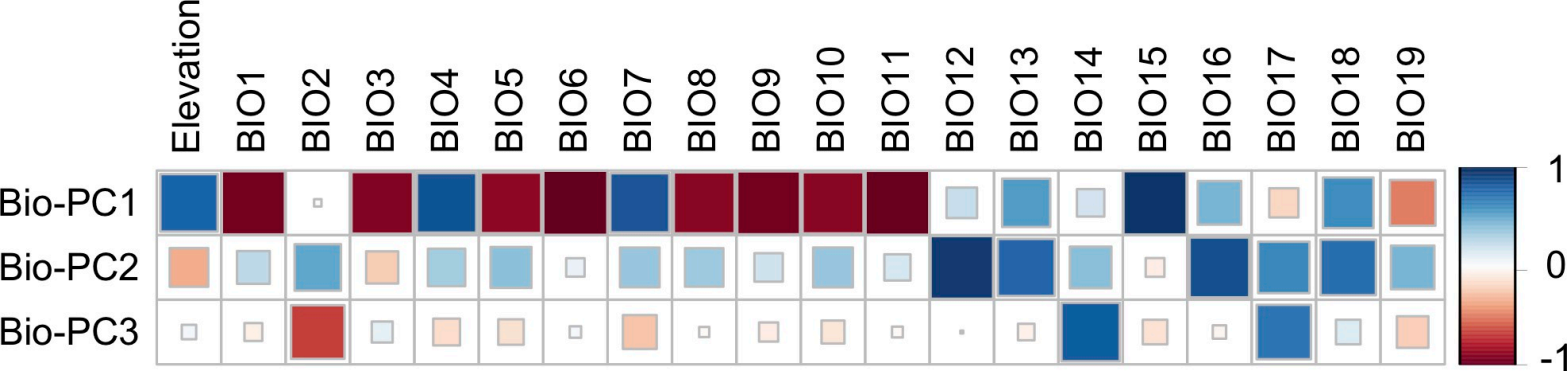
Correlation between the first three principal components (Bio-PC1–3), elevation, and environmental variables (BIO1–BIO19). Positive correlations are represented in blue and negative correlations in red. Color intensity and size of squares are proportional to the correlation coefficients (*p* < 0.01). Bioclimatic variables (BIO1-BIO19) are described in S1 Table.

Maps of the 19 bioclimatic variables extracted from the WorldClim.2 database (www.worldclim.org) revealed spatial patterns of temperature and precipitation across the collection sites (S1 and S2 Figs). The first three Bio-PCs explained 89.8% of the total variance. Bio-PC1 (56.9%) was largely correlated with temperature variables and elevation, which is consistent with the predominantly north-south distribution of the collection sites (Fig 3), and the elevation gradient that ranges from 250 to 1300 m at the base of the foothills of the Andes [4]. Bio-PC2 (23.6%) was primarily correlated with precipitation variables, most likely reflecting the rainfall gradient across the west-east sampling points, from the humid Yungas to the Dry Chaco.

### Genotype-environment associations

**Fig 4:**
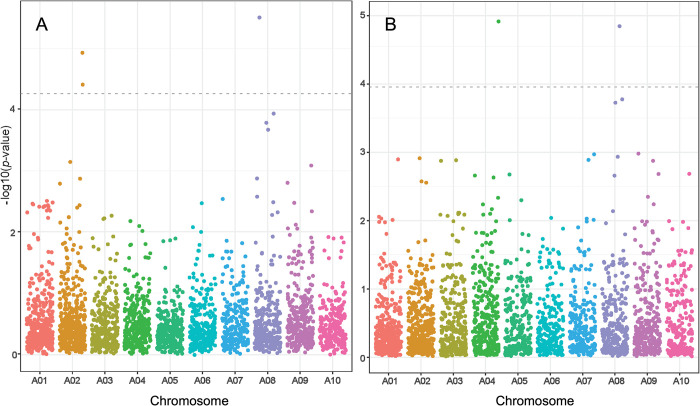
Manhattan plot for SNP *p*-values from the LFMM2 analysis. (A) First principal component (Bio-PC1). (B) Second principal component (Bio-PC2). Single Nucleotide Polymorphisms that were significant after adjusting for multiple testing are above the grey dotted line.

The LFMM2 analysis detected significant genotype-environment associations (*q <* 0.05) indicating genomic regions likely to be involved in adaptation to climate variation. For Bio-PC1, 43 SNPs were identified (*p* < 0.01), of which 11 were highly significant (*p* < 0.001), and three remained significant after adjustment for multiple testing (*q* < 0.05) (Fig 4A, S3 Table). They mapped to two different regions of the *A*. *duranensis* reference genome, one on chromosome A02 (SNP loci AX-147214002 and AX-176799664), and the other on chromosome A08 (AX-177642740). Single nucleotide polymorphism markers on chromosome A02 were within or near a Cytochrome P450 annotated gene (S3 Table). On chromosome A08, the SNP AX-177642740 mapped less than 0.27 Mb from a disease resistance protein (TIR-NBS-LRR class) and close (< 0.5 Mb) to a WRKY transcription factor gene (S3 Table). SNP alleles in AX-147214002 (A02) and AX-177642740 (A08) were specific to southern accessions (group 5 in Fig 1) collected at high elevation (> 900 m) in Salta and Jujuy (Argentina). They included accessions PI 468197, PI 468200, PI 468198, PI 497264, PI 497263, PI 475884, PI 475886, PI 475887, PI 497265, and PI 262133, all showing little (< 20%) or no admixed individuals. One exception for AX-147214002 was accession PI 497269, which carried the same allele as those collected at high elevation (> 900 m), but it was collected at 450 m. This accession contained genetic components from different groups, including group 5, suggesting that the AX-147214002 allele originated from the group of accessions from high elevation (Fig 2, 1–5). As elevation was negatively correlated with temperature-related bioclimatic variables (average *r* = 0.88, *p*-value < 0.01), comparatively lower values (°C) were observed for BIO1, BIO5–6, and BIO8–11 at their collection sites (S1 and S2 Figs). Consequently, this germplasm might have the potential to be utilized in introgression breeding as a source of adaptive allelic variation for cold tolerance.

For Bio-PC2, 36 SNPs showed significant genotype-environment associations (*p* < 0.01), four of these SNPs were highly significant (*p* < 0.001), and two remained significant after adjustment for multiple testing (*q* < 0.05). They mapped on chromosome A04 (AX-176796447) and A08 (AX-147229475) (Fig 4B, S3 Table). The SNP on chromosome A04 mapped to an intergenic region, while AX-147229454 on A08, mapped within the ER lumen protein retaining receptor family protein gene (S3 Table).

### Early and late leaf spot evaluation and genotype-disease response associations

**Fig 5:**
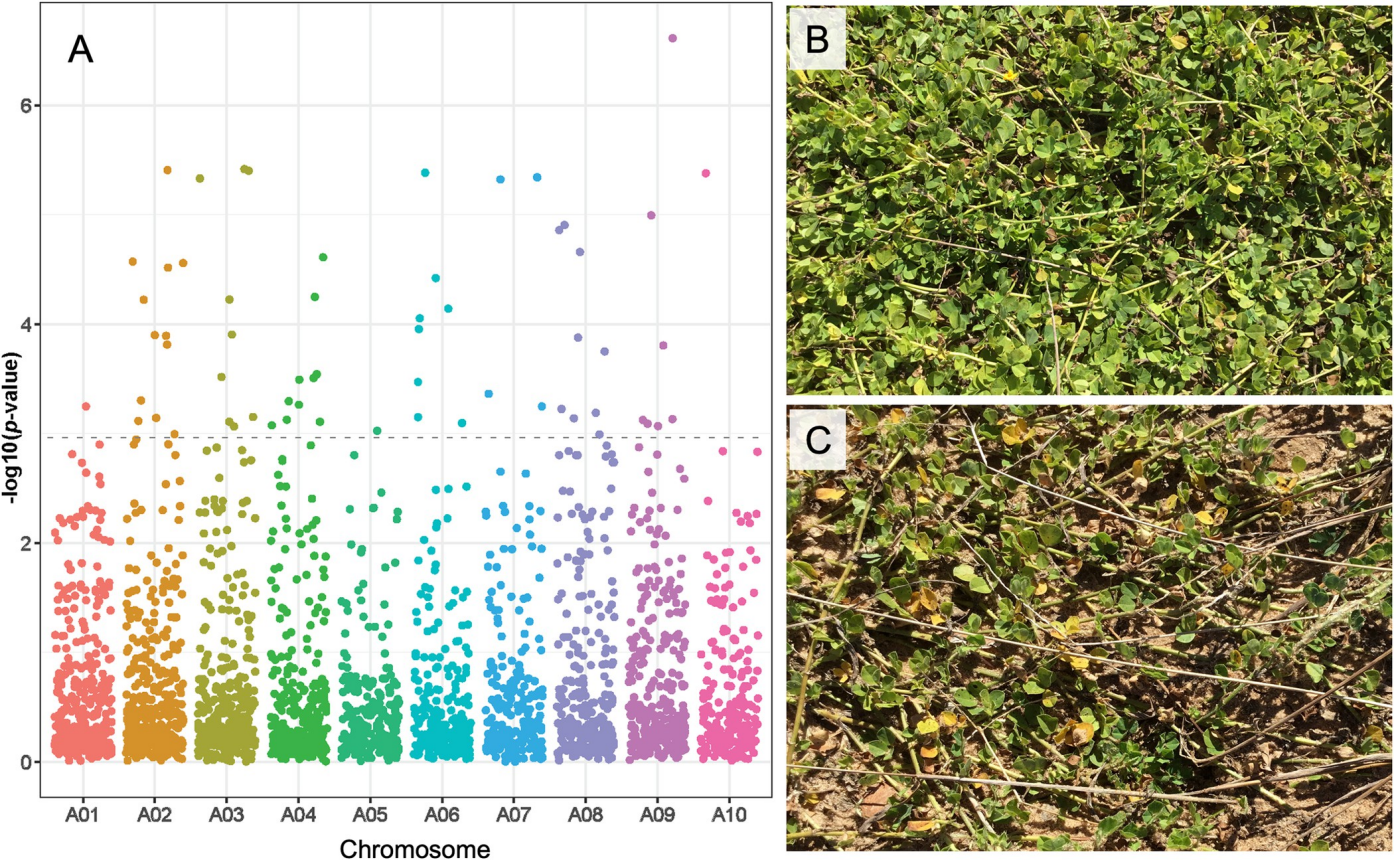
Evaluation of Leaf spot resistance of final defoliation (FD). (A) Manhattan plot for SNP *p*-values from the LFMM2 analysis. SNPs that remained significant after adjusting for multiple testing (*q* < 0.05) are above the gray dot line. (B) Accession PI 468197. (C) Accession PI 497268.

Environmental conditions during the two years of disease evaluations (2017–2018) were favorable for ELS and LLS infestation, as evidenced by the severe leaf spot symptoms observed in both susceptible and resistant control plots (S4 Table). Disease resistance variables across accessions exhibited a broad range of phenotypic variation, ranging from 0.0 to 6.1 (ELC), 0.8 to 13.4 (LLC), and 2.0 to 6.0 (FD) (S4 Table). Differences between accessions for all three disease variables were highly significant (*p* < 0.001), FD rating and ELC also showed significant year effect (*p* < 0.05). For final defoliation, the lowest levels were registered for PI 497264, PI 468324, PI 468319, and PI 468197, with an average FD ~ 2.0 (*p* < 0.001), while the highest levels were observed for PI 468201 and PI 468202, with an average FD ~ 6.0 (*p* < 0.001), followed by PI 497268 (FD ~5.0, *p* < 0.001) (Fig 5B and 5C). Accessions PI 468201 and PI 468202 are those with the highest levels of similarity to the A subgenome of *A*. *hypogaea* [46,47], which could be a potential explanation for the relatively low levels of resistance found in cultivated peanut. There are no previous reports of ELS and LLS field screening under natural infestation for the *A*. *duranensis* accessions of the USDA peanut germplasm collection evaluated in this study. Differential levels of LLS resistance have been previously identified in *A*. *duranensis* using detached leaf assays [10,30]. In such assays, components of resistance such as lesion diameter and amount of sporulation have shown consistency when used for disease screening across different environments and could be informative for evaluating genotypes for resistance to leaf spots in peanut [36]. However, defoliation rates cannot be determined by detached leave assays [49].

The analysis of ELS and LLS disease variables using the latent factor mixed model (LFMM2) revealed statistically significant genomic associations (*q* < 0.05). For final defoliation rating (FD), 185 significant SNPs were detected (*p* < 0.01), 61 of these remained significant after adjustment for multiple testing (*q* < 0.05), 20 of which were significant at *q* < 0.01 (Fig 5A, S3 Table). For the other two disease variables (ELC and LLC), the LFMM2 analysis identified 50 (LLC) and 38 (ELC) significant associations (*p* < 0.01), of which 11 SNPs (LLC) were significant at *p* < 0.001. None of these SNPs remained significant after Benjamini-Hochberg corrections. Nevertheless, 11 significant SNPs located on chromosomes A02, A03, A06, A08, and A10 were commonly detected for LLC (*p* < 0.001) and FD (*q* < 0.01) (S3 Table).

Most statistically significant SNPs associated with FD (*q* < 0.01) mapped to six different genomic regions distributed among five chromosomes, A02, A03, A04, A06, and A08. A few other SNPs mapped to single genes or loci on chromosomes A07, A09, and A10. Single nucleotide polymorphisms on chromosomes A02, A03, A04, and A09, were within annotated genes, or in nearby regions harboring genes involved in plant defense responses against abiotic and biotic stress (S3 Table). On chromosome A02, SNPs mapped to two genomic regions, one at position 0.3–4.1 Mb (upper end) and other at position 77.2–77.4 Mb (lower end). The region at the upper end harbors a cluster of Nucleotide Binding-Leucine-Rich Repeat (NB-LRR) disease resistance protein genes. While the region at the lower end is relatively close (< 2 Mb) to a hotspot of disease resistance protein genes (TIR-NBS-LRR class) at position 77.1–77.7 Mb. Regression of the FD mean values on the genotype of these SNPs explained between 11.4% and 35.9% of the phenotypic variance (*p* < 0.001) (S3 Table). A post-hoc Tukey-Kramer test indicated statistically significant differences in FD mean values between genotypes groups at the SNP positions (*p* < 0.001). Alleles present in resistant individuals (FD < 2.5) were absent in susceptible individuals (FD > 4.75), and *vice versa* (S2 Table).

On chromosome A03, statistically significant SNPs mapped at position 131.0–133 Mb (lower end), and relatively close (~ 0.5–2.0 Mb) to a region containing genes associated with plant responses to abiotic and biotic stress. These included a gene cluster of the cytochrome P450 superfamily, a zinc finger CCCH domain-containing protein, and a disease resistance protein (TIR-NBS-LRR class) gene. Regression of the FD mean values on the genotype of these SNPs explained between 26.7% and 32.1% of the phenotypic variance (*p* < 0.001). The Tukey-Kramer test of pairwise comparisons further showed significant differences of FD mean scores among groups of genotypes (*p* < 0.001). Alleles present in all individuals with FD mean values < 2.5 were absent in all individuals with FD mean values > 4.75, and *vice versa* (S2 Table).

These A02 and A03 chromosome regions have been consistently associated with LLS resistance in peanut breeding lines and cultivars carrying similar segments introgressed from the wild diploid species *A*. *cardenasii* Krapov. & W.C. Gregory (accession GKP 10017) [49–53]. The A03 region of *A*. *cardenasii* has also been associated with resistance to ELS [51] and rust fungal disease (*Puccinia arachidis* Speg.) [50,52,53]. Distinct patterns of introgression into various genetic backgrounds conferred variable levels of leaf spot and rust resistance [49,50]. Comparative analysis of syntenic regions in *A*. *cardenasii* and the *A*. *duranensis* reference genome (accession V14167) further revealed a larger number of disease resistance genes (NB-LRR) in the chromosome segments of A02 (upper end) and A03 (lower end) of *A*. *cardenasii* compared to *A*. *duranensis* [50]. While the biological significance of these differences remains to be elucidated, the copy number variation of NBS-LRR genes in the diverse germplasm of *A*. *duranensis* merit further investigation. Structural variations such as gene presence-absence and copy number variation have often been associated with disease resistance in plants [54].

A different source of LLS resistance associated with chromosomes A02, A04, and A06 was detected in peanut introgression lines carrying wild chromosome segments from *A*. *stenosperma* [24]. Two major QTLs mapped on chromosomes A02 and A06 at positions 86.0–92.0 Mb and 93.0–110.0 Mb, respectively [24]. These chromosome positions are similar to those found in the current study (S3 Table). The QTL detected on chromosome A04 of *A*. *stenosperma* (0–7.0 Mb) [24] is also comparable to one of the two A04 chromosome regions detected here (1.7–6.7 Mb). Furthermore, the region at the end of chromosome A02 has been associated with resistance to peanut smut *Thecaphora frezii* Carranza & Lindquist [55] and root-knot nematode (PRKN) *Meloidogyne arenaria* [56]. Collectively, the detection of candidate SNPs and genomic regions in this study demonstrates the intraspecific genetic variation for leaf spot resistance in *A*. *duranensis*.

### Overlap between environmental and disease response variables

In addition to common SNPs and genomic regions between FD and LLC, the overall LFMM2 association analysis detected seven statistically significant SNPs that overlapped between two or more environmental and disease-related variables. They mapped to five different genomic regions distributed among chromosomes A02, A03, A08, and A09 (S3 Table). Two SNPs, AX-147212902 on chromosome A02 and AX-177640526 on chromosome A08 were common to Bio-PC1, FD, and LLC variables. The SNP AX-147212902 mapped to the lower end of chromosome A02 (77.2–77.4 Mb) as described earlier. The SNP AX-177640526 mapped to a gene-rich region including phytosulfokine 4 precursor (Leu-rich repeat (LRR)-RLK) and Zinc finger GRF-type protein genes, both characterized by their role in plant growth, development, and stress response. Two other SNPs, AX-176820714 on chromosome A02 and AX-147230302 on chromosome A08, were shared between precipitation-related variables (Bio-PC2) and FD. The SNP AX-176820714 is in a gene-rich region of disease resistance and stress-related genes, including LRR and NB-ARC domain disease resistance protein genes (Aradu.U5EC8, Aradu.E3TX0), disease resistance protein (TIR-NBS-LRR class, Aradu.IL2CV), and a cluster of stress up-regulated Nod 19 protein genes (e.g., Aradu.MD540, Aradu.AZ0E5 Aradu.U3JJM). The latest related to plant response to abiotic and biotic oxidative stress [57]. Co-localized genomic regions potentially involved in leaf spot resistance and adaptation to different environments will enable the simultaneous introgression of favorable alleles from wild *A*. *duranensis* into cultivated peanut.

## Conclusions

While *A*. *duranensis* has been recognized in the literature as a source of genetic diversity for disease resistance and stress tolerance, the unique approach used in the present study indicates that the most promising sources of resistance within this species have yet to be explored. The analysis of natural populations using a landscape genomic approach revealed candidate SNP loci and genomic regions associated with leaf spot diseases and environmental adaptations. These results were supported by statistically significant genotype-environment associations, predominantly temperature-related bioclimatic variables. Furthermore, field-based evaluations combined with association analyses for ELS and LLS, led to the identification of genomic regions and candidate SNPs associated with resistance to the diseases. Finally, we report new sources of *A*. *duranensis* germplasm that have the potential to be utilized in introgression breeding for abiotic stress tolerance and resistance to leaf spot diseases.

## Supporting information

S1 Fig Temperature-related bioclimatic variables (BIO1-BIO11) across the accession collection sites. Source: https://worldclim.org. Descriptions are listed in S1 Table.
(PDF)

S2 Fig Precipitation-related bioclimatic variables (BIO12-BIO19) across the accession collection sites. Source: https://worldclim.org. Descriptions are listed in S1 Table.
(PDF)

S1 Table WorldClim2 bioclimatic variables and their descriptions. Source: https://worldclim.org.
(XLSX)

S2 Table Genotype scores of 32 PI accessions and 2810 SNPs of the Axiom Arachis2 array. (XLSX)

S3 Table Statistically significant SNPs (*p* < 0.01) detected in the analysis for Bio-PC1, Bio-PC2, and FD variables. Chromosome, position, gene model, location, and description data are based on the *Arachis duranensis* reference genome (www.peanutbase.org).
(XLSX)

S4 Table Disease scores for ELS and LLS. (XLSX)
