# Supplementary figures and images for: Genetic diversity, disease resistance, and environmental adaptation of Arachis duranensis L.: New insights from landscape genomics
Source: PLoS One. 2024 Apr 16;19(4):e0299992. doi: 10.1371/journal.pone.0299992 (PMC11020403; doi:10.1371/journal.pone.0299992)

**BIO1**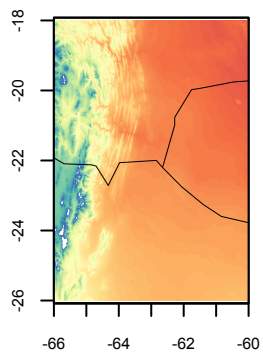**BIO2**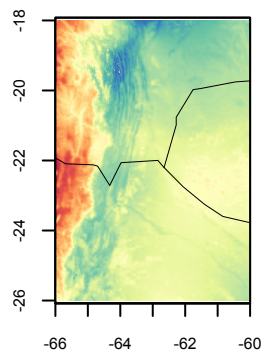**BIO3**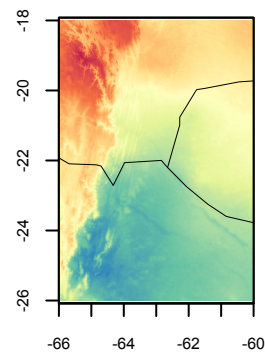**BIO4**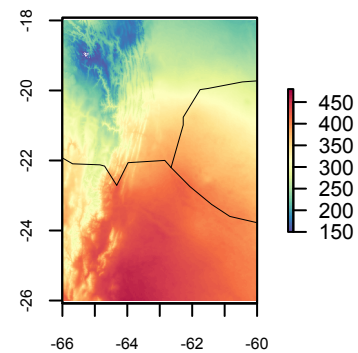**BIO5**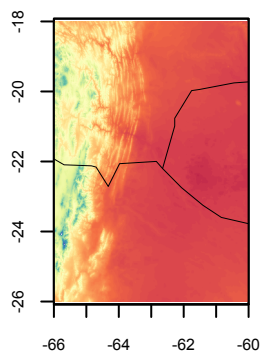**BIO6**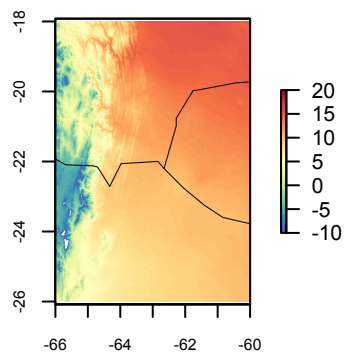**BIO7**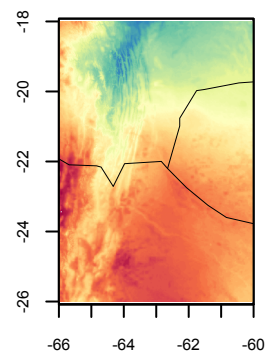**BIO8**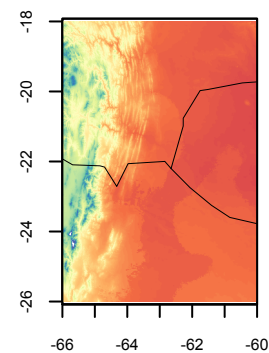**BIO9**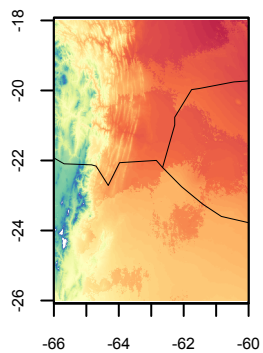**BIO10**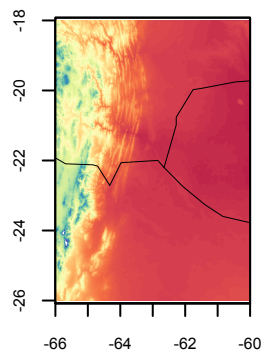**BIO11**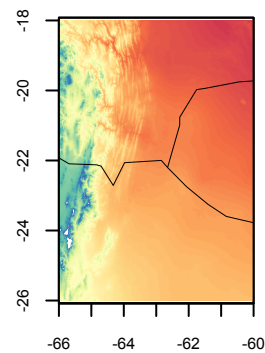

Supplement: S1 Fig — Source: https://worldclim.org. Descriptions are listed in S1 Table. (PDF) [file pone.0299992.s001.pdf]

**BIO12**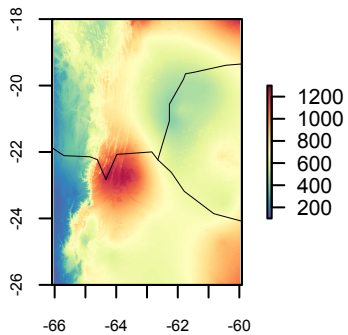**BIO13**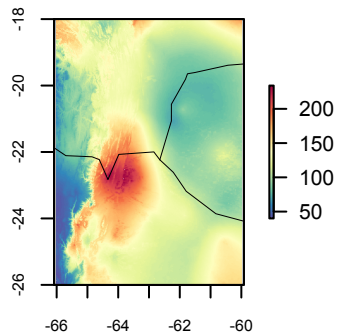**BIO14**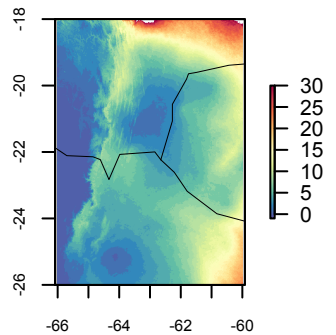**BIO15**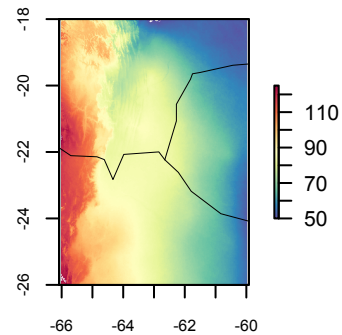**BIO16**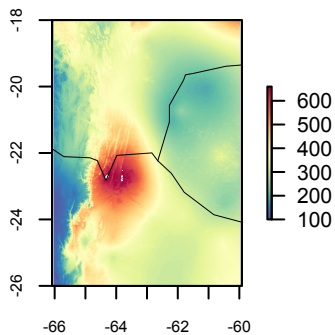**BIO17**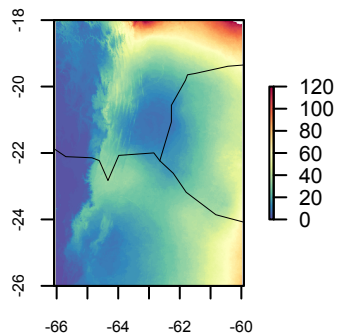**BIO18**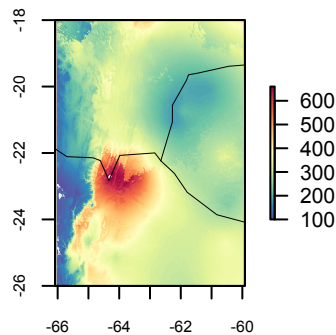**BIO19**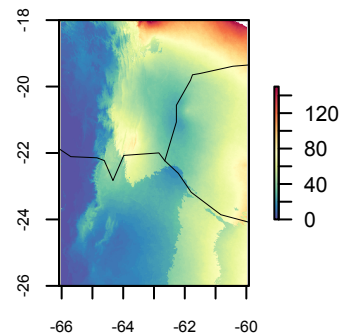

Supplement: S2 Fig — Source: https://worldclim.org. Descriptions are listed in S1 Table. (PDF) [file pone.0299992.s002.pdf]
